# Reclassifying congruence in wellbeing: a systematic review and alignment framework

**DOI:** 10.3389/fpsyg.2026.1859907

**Published:** 2026-10-01

**Authors:** Brigid McEvoy

**Affiliations:** Department of Psychology, University of Windsor, Windsor, ON, Canada

**Keywords:** alignment, self-determination theory, systematic review, wellbeing, within-person processes

## Abstract

Research on wellbeing has increasingly shifted toward within-person and short-timescale processes, yet remains conceptually fragmented across motivational, relational, and value-based literatures. Constructs reflecting congruence between internal standards (e.g., needs, values, identity) and lived experience are widely studied but rarely integrated. This systematic review aimed to synthesize how alignment-related processes are defined, operationalized, and empirically linked to momentary wellbeing and short-term disruption/recovery within individuals. A PRISMA-informed systematic review was conducted across major psychological and interdisciplinary databases. Studies were included if they examined motivational, moral/value, or relational alignment processes and assessed momentary or short-term wellbeing outcomes using within-person, repeated-measures, or theoretically relevant designs. A total of 130 empirical and conceptual studies met inclusion criteria. Data were extracted using a structured protocol and synthesized using interpretive conceptual analysis. Findings indicate that the literature does not converge on a single construct of alignment, but rather reflects multiple functionally distinct processes involving correspondence between internal orientations and lived experience. These processes can be organized into (a) indicators of correspondence (e.g., autonomous motivation, value congruence), (b) contextual conditions shaping their expression (e.g., autonomy support), and (c) mechanisms linking alignment to outcomes (e.g., psychological need satisfaction). Empirical evidence is strongest at momentary and same-day timescales, with consistent within-person associations with affect, vitality, and engagement. Evidence for lagged or extended temporal dynamics is comparatively limited. Support is most robust for motivational processes, moderate for relational processes, and comparatively weak for value-based processes, particularly in temporally sensitive designs. Alignment is best understood as a multi-process, multi-domain framework rather than a unified construct. This review clarifies conceptual overlap, identifies asymmetries in empirical support, and delineates conditions under which dynamic models of wellbeing can be more rigorously tested. Findings provide a foundation for developing process-based, within-person models of flourishing.

## Reclassifying congruence in wellbeing

Research on wellbeing has increasingly shifted toward understanding short-timescale, within-person processes, yet remains conceptually fragmented across psychological literatures. Flourishing has been variously conceptualized in terms of psychological need satisfaction, value alignment, and affective or experiential states (Ryan and Deci, 2000; Martela and Steger, 2016, Martela et al., 2018). Although each perspective captures important aspects of wellbeing, these frameworks are often developed in parallel and treated as interchangeable despite reflecting distinct theoretical assumptions. This has resulted in substantial conceptual fragmentation, limiting the development of cumulative and coherent models of wellbeing.

A recurring theme across these literatures is the notion of congruence between internal orientations, such as needs, values, and identity, and behavior or lived experience. This idea is reflected in constructs such as autonomous motivation and need satisfaction within self-determination theory (Ryan and Deci, 2000), value congruence and self-discrepancy within moral and identity-based frameworks (Higgins, 1987; Aquino and Reed, 2002), and autonomy support and responsiveness within relational models (Deci et al., 1989; Reis and Gable, 2015; Grant, 2008). Despite this conceptual convergence, these constructs are typically examined in isolation and operationalized using different terminologies and measurement approaches, obscuring their potential relationships.

At the same time, a growing body of research indicates that wellbeing is not a stable trait, but a dynamic process that fluctuates within individuals over short timescales. Evidence from daily diary and experience sampling studies demonstrates meaningful within-person variation in affect, vitality, engagement, and related outcomes (Houben et al., 2015; Ilies et al., 2017; Sosin and Neubauer, 2024). These findings challenge traditional trait-based models and suggest that wellbeing emerges through ongoing interactions between individuals and their environments. However, existing theoretical frameworks have not fully incorporated these temporal dynamics, nor have they adequately specified how short-term processes such as disruption and recovery unfold.

This limitation is increasingly consequential because intensive longitudinal, daily diary, and ecological momentary assessment studies reveal substantial within-person variability in motivation, affect, need fulfillment, authenticity, social connection, and wellbeing (Bolger et al., 2003; Reis and Gable, 2000; Ryan and Deci, 2017). These findings suggest that flourishing may be better understood as a dynamic process than a stable condition. Yet few syntheses have examined how alignment-related processes are maintained, disrupted, and potentially restored across motivational, relational, and moral/value domains. Importantly, the present review does not assume that all alignment-related constructs fluctuate to the same degree. Constructs such as moral identity, value orientation, and self-concordance may function as relatively stable internal reference points, whereas the enactment, expression, fulfillment, or frustration of these orientations can vary substantially across situations and time. Accordingly, the review focuses on short-timescale variation in correspondence between internal orientations and lived experience rather than assuming equivalent short-term variability in all underlying constructs.

A key limitation underlying this fragmentation is the lack of differentiation between conceptually related constructs. For example, psychological need satisfaction is often treated as an indicator of alignment, despite functioning more consistently as a downstream mechanism linking alignment-related processes to wellbeing (Ryan and Deci, 2000; Reis et al., 2000).

Similarly, relational constructs such as autonomy support are frequently modeled as independent predictors, rather than as contextual conditions shaping the enactment of internally endorsed behavior (Deci et al., 1989; Legate et al., 2017). Value-based constructs, including value congruence and moral identity, represent a conceptually relevant domain but remain comparatively underdeveloped in temporally sensitive research designs (Sheldon and Krieger, 2014; Prentice et al., 2020). These patterns contribute to conceptual overlap, redundancy, and inconsistent interpretation across studies.

For the purposes of this review, correspondence refers to the degree to which behavior, goals, relationships, or contexts fit, support, or express an individual’s internal orientations, including motives, needs, values, and identities (Ryan and Deci, 2000, 2017; Sheldon and Elliot, 1999; Reis et al., 2000; Kristof, 1996). Alignment refers to experienced or enacted correspondence, such as self-endorsed action, value enactment, or authentic self-expression (Sheldon and Elliot, 1999; Kernis and Goldman, 2006; Prentice et al., 2020). Disruption refers to breakdowns or mismatches in these relationships, including need frustration, self-discrepancy, value incongruence, relational conflict, or environmental misfit (Higgins, 1987; Ryan and Deci, 2000, 2017; Legate et al., 2017). Restoration refers to processes through which disrupted correspondence is re-established, compensated for, or reorganized, including repair, crafting, recovery, and behavioral adjustment (van Hooff and Geurts, 2014; Laporte et al., 2022; Xue et al., 2022; Kujanpää and Olafsen, 2024). Although these constructs originate from different theoretical traditions, they share a common focus on correspondence between internal orientations and lived experience. Autonomous motivation, self-concordance, value congruence, authenticity, person-environment fit, and related constructs differ in their substantive emphasis, yet each addresses whether thoughts, goals, behaviors, relationships, or contexts are experienced as consistent with psychologically significant aspects of the self (Ryan and Deci, 2000, 2017; Sheldon and Elliot, 1999; Kristof, 1996; Reis et al., 2000; Prentice et al., 2020).

To address these issues, the present review introduces alignment as a conceptual framework for organizing congruence-based processes according to their functional role in wellbeing. Alignment is not conceptualized as a standalone construct or explanatory mechanism; rather, it provides a structured way of mapping relationships among constructs that capture correspondence between internal orientations (e.g., motives, values, needs) and lived experience (Deci and Ryan, 2000; Higgins, 1987; Sheldon and Elliot, 1999; Reis et al., 2000). Within this framework, constructs are differentiated into (a) indicators of correspondence (e.g., autonomous motivation, value congruence), (b) contextual conditions shaping their expression (e.g., autonomy support, responsiveness), and (c) mechanisms linking these processes to wellbeing outcomes (e.g., psychological need satisfaction). This distinction matters because construct proliferation is often treated as a problem of redundant labels. The present review suggests a different possibility: some constructs may overlap conceptually while differing functionally. For example, one construct may describe a contextual condition that enables alignment, another may index alignment itself, another may explain why alignment predicts wellbeing, and another may capture disruption or restoration after alignment breaks down. Existing theories typically specify these relations within particular domains, such as motivation, relationships, values, or person–environment fit. The present review examines whether similar functional roles recur across these domains and whether this perspective can help organize a fragmented literature.

This functional distinction addresses a central limitation in the literature by clarifying how constructs that are often treated as interchangeable may instead occupy different roles within a shared process structure. Without such differentiation, constructs that occupy theoretically distinct positions within explanatory models may be treated as interchangeable predictors, obscuring the mechanisms through which correspondence influences wellbeing. It also provides a basis for evaluating whether processes across motivational, relational, and value-based domains can be integrated within a dynamic, within-person account of wellbeing. Current evidence suggests that support is strongest for motivational processes, moderate for relational processes, and comparatively limited for value-based processes, particularly in intensive longitudinal designs (Sosin and Neubauer, 2024; Bachmann et al., 2024; Legate et al., 2017).

Accordingly, the present study conducts a systematic review and conceptual synthesis to examine how alignment-related processes are defined, operationalized, and empirically linked to momentary wellbeing and short-term disruption and recovery. Rather than proposing a unified construct, the goal is to organize existing constructs, identify sources of conceptual fragmentation, and specify conditions under which integration can be more rigorously tested. By doing so, this review aims to advance a more precise, process-oriented understanding of wellbeing grounded in within-person dynamics.

## Research questions

### Conceptualization

How are motivational, moral/value, and relational alignment processes defined and operationalized across psychological literatures?

### Temporal structure

What empirical evidence links these alignment-related processes to momentary wellbeing outcomes (e.g., vitality, engagement, affect)?

### Mechanisms

How do existing theories conceptualize short-term disruption and recovery at within-person timescales, and to what extent are motivational, moral/value, and relational alignment processes integrated within these models?

## Method

### Study design

This review employed a PRISMA-informed systematic methodology combined with an interpretive conceptual synthesis to examine congruence-based (alignment) processes in relation to short-timescale wellbeing. This design was selected to address a literature characterized by substantial construct heterogeneity and theoretical fragmentation, in which constructs are variably defined and distributed across motivational, relational, and value-based domains. In such contexts, the primary objective is not effect-size aggregation but the systematic identification, classification, and integration of shared conceptual and relational structures across studies (Dixon-Woods et al., 2005; Grant and Booth, 2009; Page et al., 2021). Interpretive synthesis is particularly appropriate where constructs overlap conceptually but differ in terminology and operationalization, and where theoretical integration is required to clarify underlying processes (Dixon-Woods et al., 2005).

### Search strategy

A systematic literature search was conducted across PsycINFO, Scopus, JSTOR, PsycArticles, Web of Science, ERIC, Academic Search Complete, Sociological Abstracts, and ProQuest databases. The search strategy was structured around three core conceptual domains:

(1) congruence-based constructs (e.g., autonomous motivation, self-concordance, value congruence, self-discrepancy, autonomy support), (2) wellbeing outcomes (e.g., affect, vitality, engagement), and (3) short-timescale or within-person designs (e.g., experience sampling, ecological momentary assessment, daily diary).

Search strings were developed using Boolean operators and proximity terms to maximize sensitivity while maintaining conceptual specificity. Consistent with the review protocol, the search was designed to capture conceptually relevant constructs across disciplinary boundaries while minimizing retrieval of studies focused exclusively on trait or global wellbeing without temporal resolution. Search strategies were iteratively refined to account for variation in terminology across motivational, relational, and value-based literatures.

Database-specific search strings, search dates, and retrieval counts are reported in Appendix A. In addition to database searches, backward and forward citation tracking was conducted to identify additional relevant studies, and supplementary records were included where they met eligibility criteria (Grant and Booth, 2009; Page et al., 2021). To reduce publication bias and improve search comprehensiveness, grey literature sources were also incorporated through ProQuest Dissertations and Theses, supplemented by citation tracking procedures and targeted searches of reference lists for relevant conceptual and empirical work.

Eligibility criteria were structured according to a modified PICOS framework. Population included adolescents and adults examined in motivational, relational, or value-based contexts.

Phenomena of interest consisted of alignment-related, congruence-based, or correspondence-based constructs. Comparator conditions were not required due to the observational and conceptual nature of much of the literature. Outcomes included momentary, daily, or short-term indicators of wellbeing, vitality, engagement, affect, recovery, or related psychological functioning. Eligible study designs included intensive longitudinal, diary, EMA, repeated-measures, observational, and theoretically relevant conceptual studies.

### Screening and study selection

Study selection followed a two-stage screening process consistent with PRISMA guidelines (Page et al., 2021). In the first stage, titles and abstracts were screened for relevance to congruence-based processes and short-timescale wellbeing outcomes. In the second stage, full-text articles were assessed against predefined eligibility criteria.

Studies were included if they examined relationships involving correspondence or discrepancy between internal orientations (e.g., motives, needs, values) and behavior, goals, or contextual conditions, and if they assessed outcomes at momentary, daily, or short-term timescales using designs sensitive to within-person variation (e.g., experience sampling, diary, repeated-measures, or short-lag designs). Studies were excluded if they focused exclusively on trait-level or global wellbeing, lacked an explicit congruence structure, or did not involve correspondence-based processes.

A PRISMA flow diagram summarizing the identification, screening, eligibility, and inclusion process is presented in Figure 1.

**Figure 1:**
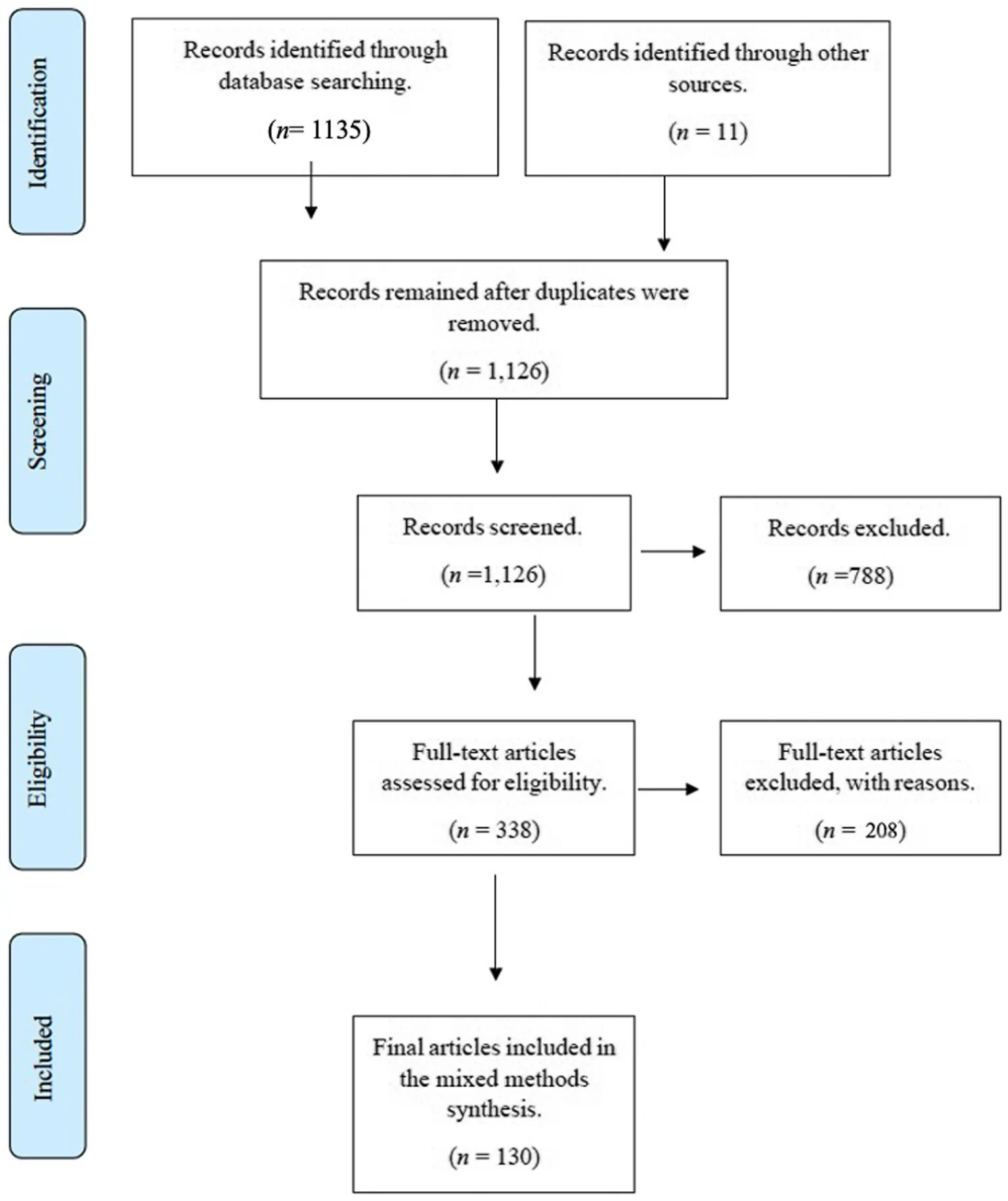
PRISMA flow diagram.

Primary reasons for exclusion at the full-text eligibility stage are summarized in Appendix E. Categories included the absence of an alignment-related construct, lack of a short-timescale or within-person outcome, exclusive focus on trait-level wellbeing, insufficient relevance to the review questions, and methodological or reporting limitations.

### Data extraction and coding

Data extraction and coding were conducted using a structured framework designed to ensure consistency, transparency, and reproducibility. Extraction was performed at both the study and construct level, capturing study characteristics and conceptual features necessary for cross-study comparison.

Extracted variables included study design, sample characteristics, domain classification (motivational, relational, value-based), construct family, temporal structure, and proposed mechanisms. Constructs were additionally classified according to their functional role within the alignment framework, including whether they operated as indicators of correspondence, mechanisms linking processes to outcomes, or contextual conditions influencing alignment.

Because the review involved interpretive classification across conceptually overlapping literatures, coding procedures were iterative. Initial coding categories were developed based on definitional features and refined through repeated application across studies. Ambiguous cases were evaluated using explicit decision rules, including prioritization of the construct’s dominant functional role, consistency with within-person designs, and directionality within empirical models.

To enhance consistency, a subset of studies was independently reviewed during development of the coding framework, and discrepancies were resolved through rule-based discussion and refinement of category definitions. An audit trail documenting coding decisions and category revisions was maintained throughout, consistent with best practices in interpretive synthesis (Dixon-Woods et al., 2005). Given the interpretive and theory-driven nature of the coding framework, formal inter-rater reliability statistics were not calculated. Instead, reliability was ensured through an iterative consensus-based coding process. A subset of studies was independently reviewed during development of the coding framework, with discrepancies resolved through structured discussion and refinement of category definitions. Coding decisions were guided by explicit decision rules, and an audit trail was maintained to document classification decisions and revisions. This approach is consistent with best practices in interpretive synthesis, where the objective is conceptual clarity and theoretical integration rather than categorical agreement (Dixon-Woods et al., 2005).

### Quality appraisal

Given the inclusion of heterogeneous empirical designs and theoretical contributions, no single risk-of-bias tool was appropriate. Accordingly, design-specific appraisal criteria were applied for empirical studies, and a structured theoretical relevance framework was used for conceptual papers, consistent with recommendations for mixed conceptual–empirical reviews (Grant and Booth, 2009).

Quantitative observational studies were evaluated using criteria adapted from established appraisal frameworks, focusing on sampling appropriateness, measurement validity, control of confounding variables, and statistical analysis rigor. Intensive longitudinal, diary, and experience sampling studies were assessed using these criteria in addition to design-specific considerations, including within-person sampling density, participant compliance and retention, separation of within- and between-person effects, temporal alignment between predictors and outcomes, appropriateness of lag structures, and justification of measurement timescales. These criteria reflect established methodological standards for within-person and micro-longitudinal research (Bolger and Laurenceau, 2013).

## Results

### Study selection

The study selection process followed PRISMA guidelines and is summarized in Figure 1.

A total of 1,135 records were identified through database searching, with an additional 11 records identified through supplementary sources. After removal of duplicates, 1,126 records were screened at the title and abstract level. Of these, 338 articles were retained for full-text review. Following application of predefined eligibility criteria, 130 studies were included in the final synthesis.

### Study characteristics

The included studies were characterized by a strong emphasis on intensive longitudinal designs and short-timescale processes. As shown in Table 1, the dataset was dominated by intensive longitudinal (EMA/diary) studies (*n* = 100), with fewer quantitative observational studies (*n* = 18) and theoretical or conceptual papers (*n* = 12). No qualitative studies met inclusion criteria. Methodological quality was predominantly moderate, with 34 studies rated as high quality, 92 as moderate, and 4 as low quality.

**Table 1:** Study type and quality appraisal summary.

Category	Count (n)
Intensive longitudinal (EMA/diary)	100
Quantitative observational	18
Theoretical/conceptual	12
Qualitative	0
High quality	34
Moderate quality	92
Low quality	4

Quality ratings reflect structured appraisal criteria applied across study types.

Consistent with the review’s focus, the evidence base was heavily weighted toward within-person designs, with 106 studies including within-person evidence and 55 incorporating lagged analyses (Table 2). Recovery-related processes were examined in 48 studies, indicating that restoration mechanisms were present but less frequently represented than disruption or concurrent associations.

**Table 2:** Temporal evidence characteristics.

Temporal feature	Number of studies (n)
Within-person evidence	106
Between-person evidence	57
Lagged evidence	55
Recovery-related evidence	48

Lagged evidence refers to studies testing temporal sequencing across time points. Recovery-related evidence refers to studies examining restoration or regulation processes following misalignment.

Design characteristics further indicate that studies frequently employed overlapping methodological approaches. As shown in Table 3, daily diary (*n* = 95) and EMA/ESM designs (*n* = 99) were the most common, with 108 studies incorporating short-term longitudinal structures. Cross-sectional or sparse-wave designs were less common (*n* = 23), and experimental designs (*n* = 10) and meta-analyses (*n* = 2) were comparatively rare.

**Table 3:** Distribution of studies by design and temporal structure.

Design category	Number of studies (n)
Total included studies	130
Daily diary designs	95
EMA/ESM designs	99
Short-term longitudinal designs	108
Cross-sectional (short-term)	23
Experimental/manipulation	10
Theoretical/conceptual	11
Secondary synthesis/meta-analysis	2

Categories are non-exclusive; multi-method studies contribute to multiple design categories.

Taken together, these patterns indicate that the literature is strongly oriented toward within-person, short-timescale analysis, with comparatively limited representation of experimental or long-term longitudinal designs.

### Conceptualization of alignment (RQ1)

#### Definitional structure and construct families

Conceptual synthesis indicates that the literature does not converge on a single construct but instead reflects multiple definitional families of congruence-based processes organized around shared relational logic. As shown in Table 4, the most prominent construct family was autonomous/internal endorsement (*n* = 57) within the motivational domain, followed by fit/congruence/discrepancy processes (*n* = 37) and need fulfillment/frustration (*n* = 32).

**Table 4:** Definitional families of alignment-related constructs.

Construct family	Domain	Distinct studies (n)
Autonomous/internal endorsement	Motivational	57
Need fulfillment/frustration	Motivational	32
Fit/congruence/discrepancy	Cross-domain	37
Behavioral need crafting/recovery matching	Cross-domain	20
Alignment-enabling self-insight processes	Cross-domain	26
Value orientation/internalization	Value/Moral	11
Moral identity/value enactment	Value/Moral	10
Autonomy support/need-supportive context	Relational	10
Validation/responsiveness/being understood	Relational	9
Support exchange/relational synchrony/conflict	Relational	13
Relational authenticity/disclosure/secrecy	Relational	2

Counts reflect distinct studies coded under each construct family. Categories are non-mutually exclusive.

Additional families included behavioral alignment processes (*n* = 20), alignment-enabling self-insight processes (*n* = 26), and smaller clusters within the relational and value-based domains. Relational constructs, such as autonomy support (*n* = 10) and validation/responsiveness (*n* = 9), were less frequently represented, while value-based constructs (e.g., moral identity, value enactment) were comparatively limited (*n* = 10–11).

#### Overlap and functional differentiation

Substantial conceptual overlap was observed across construct families, particularly within the motivational domain. Constructs such as autonomous motivation, self-concordance, and need satisfaction frequently co-occurred and were sometimes operationalized interchangeably, despite representing distinct functional roles. Coding procedures therefore differentiated constructs into indicators of correspondence, mechanisms, and contextual conditions, reducing redundancy and clarifying their position within a shared process structure.

#### Domain distribution

The distribution of constructs across domains was uneven. The motivational domain was the most coherent and empirically developed, followed by more diffuse relational processes and comparatively underdeveloped value-based processes. Cross-domain constructs (e.g., fit, discrepancy) further contributed to conceptual overlap, indicating that alignment-related processes span multiple theoretical traditions but are rarely integrated within a unified framework. Overall, the conceptual evidence reflects structured convergence without integration, in which constructs share underlying relational logic but differ in operationalization, domain, and functional role.

Across studies, alignment-related processes were operationalized using multiple overlapping approaches. As shown in Figure 2, endorsement-based measures (e.g., autonomous motivation) were the most frequently used operationalization (35.1%), followed by congruence-based measures (20.0%) and fit/discrepancy-based approaches (14.0%). In contrast, fewer studies directly modeled mechanisms (10.2%) or support-based processes (7.7%), indicating that the literature more often captures alignment indirectly rather than through explicitly specified process pathways.

**Figure 2:**
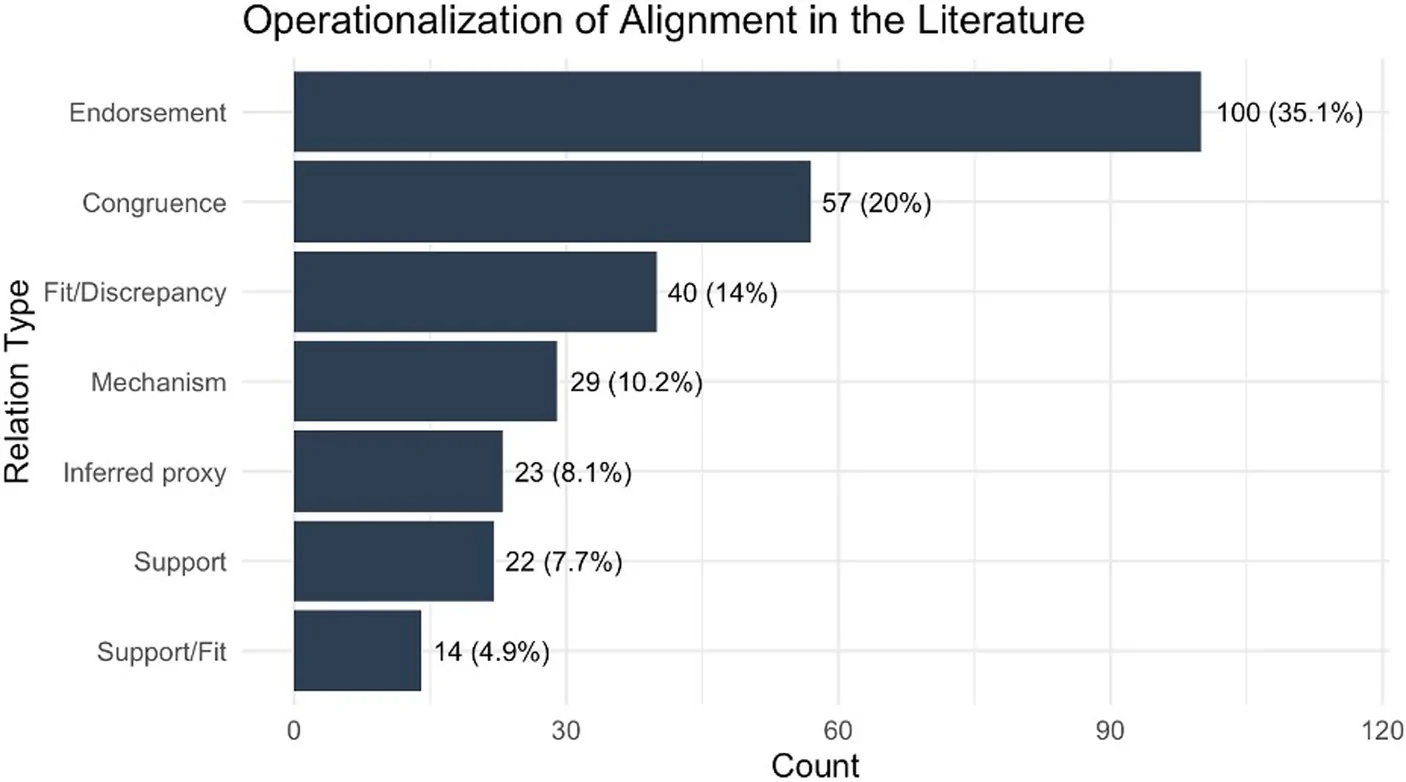
Operationalization of correspondence-based processes in the literature. Bars represent the frequency and percentage of studies using each operationalization type. Endorsement-based measures (e.g., autonomous motivation) and congruence-based measures are most common, whereas fewer studies directly model mechanisms or support-based processes. Percentages reflect proportions of the total coded observations.

### Empirical evidence (RQ2)

#### Temporal structure of evidence

The temporal distribution of evidence indicates that findings are concentrated at short timescales, particularly within-person, concurrent associations. As shown in Table 2, within-person evidence (*n* = 106) substantially exceeds lagged evidence (*n* = 55), indicating that the literature is more strongly positioned to support co-occurrence than temporal sequencing.

Consistent with this pattern, supplementary quantitative analyses indicated that short-term (within-day) observations accounted for 40.6% of coded instances, whereas lagged or temporally separated analyses accounted for only 2.0%, with the remainder represented by cross-sectional or sparsely structured designs (Appendix D).

#### Domain × timescale association

A chi-square test of independence revealed a significant association between conceptual domain and temporal structure, *χ*^2^(9) = 64.04, *p* < 0.001, Cramér’s V = 0.57, indicating that domains are unevenly represented across timescales. As illustrated in Figure 3, motivational processes are more frequently examined in momentary and daily designs, whereas relational and value-based processes are more often represented in cross-sectional or less temporally resolved studies.

**Figure 3:**
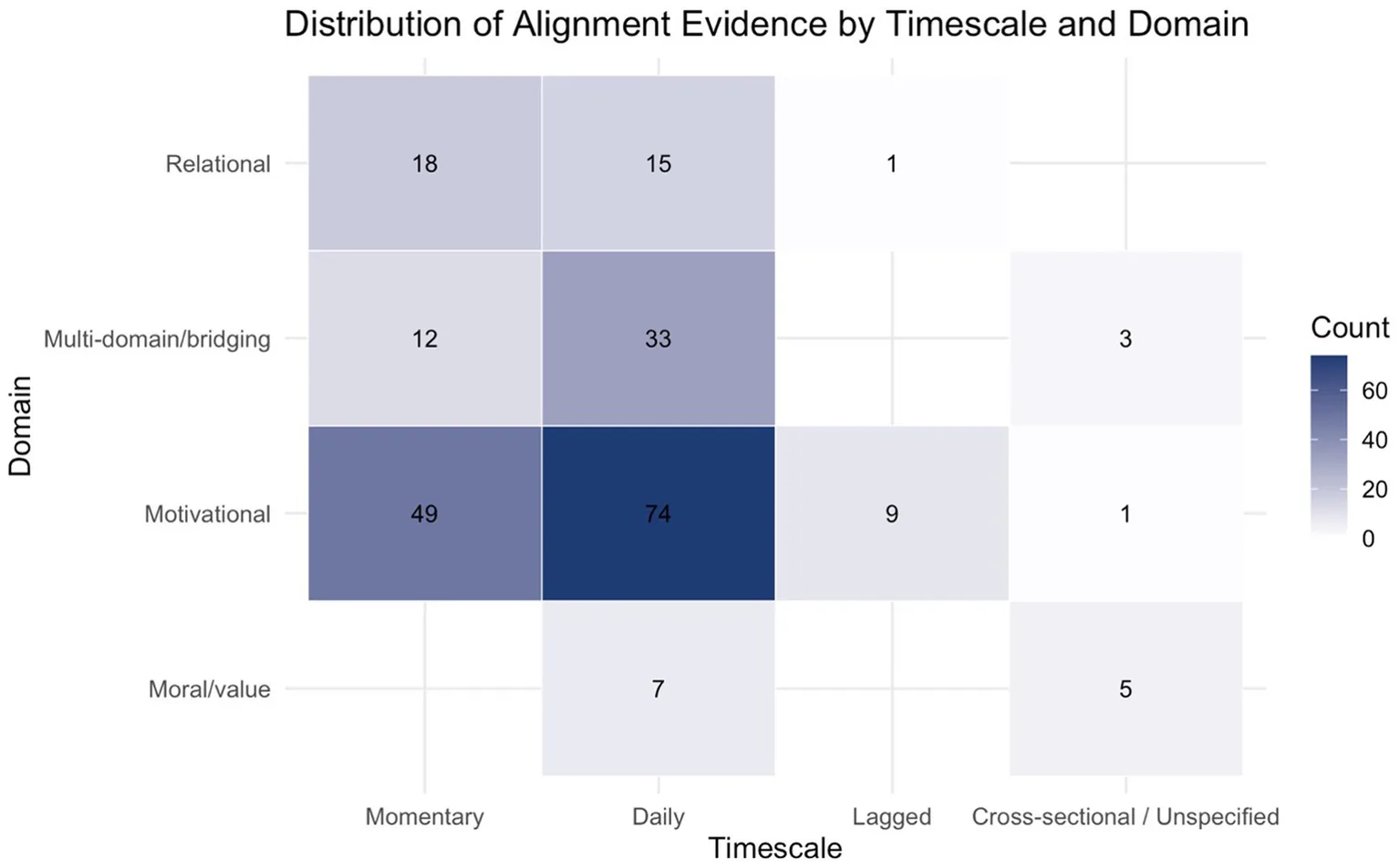
Distribution of correspondence-based processes across timescales and domains. The figure displays the frequency of coded observations across timescales (Momentary, Daily, Lagged, and Cross-sectional/Unspecified) and conceptual domains (Motivational, Relational, Moral/Value, and Multi-domain/Bridging). Darker shading indicates higher counts. Results illustrate that motivational processes are more frequently examined at momentary and daily timescales, whereas relational and value-based processes are more often represented in cross-sectional or less temporally resolved designs.

#### Outcome distribution

Across studies, alignment-related processes were most consistently associated with affective and experiential outcomes, with affect and subjective wellbeing representing the dominant outcome category. Other outcomes, including performance, recovery, and meaning-based variables, were less frequently examined, indicating that the empirical literature is concentrated on proximal experiential states rather than distal outcomes.

#### Strength of evidence across domains

Evidence strength varied systematically across domains. The motivational domain demonstrated the strongest and most consistent associations with wellbeing, particularly through within-person and short-timescale analyses. Relational processes showed moderate support, primarily as contextual influences, while value-based processes remained comparatively underdeveloped, especially in temporally sensitive designs. Overall, the empirical evidence supports short-timescale covariation between alignment-related processes and wellbeing, but provides limited support for lagged, sequential, or extended temporal dynamics.

### Mechanisms (RQ3)

#### Primary mechanistic pathways

Mechanistic analysis indicates that psychological need satisfaction and frustration represent the most consistently supported pathway linking alignment-related processes to wellbeing. As summarized in Table 5, need-based mechanisms were the most consistently represented mechanistic pathway (*n* = 32), although explicit mediation models were not consistently specified.

**Table 5:** Mechanistic evidence characteristics.

Mechanistic feature	Number of studies (n)
Studies including need-based mechanisms	32
Studies with lagged mechanistic testing	55
Studies including recovery processes	48

Need-based mechanisms refer to studies including psychological need satisfaction or frustration as pathways linking alignment-related processes to wellbeing. Counts reflect the presence of mechanisms rather than consistent or formally specified mediation models.

Categories are non-mutually exclusive, and individual studies may contribute to multiple mechanistic features. Although need-based mechanisms were the most consistently represented, they were not always explicitly modeled as mediators.

Within-person evidence suggests that motivational and contextual processes are linked to wellbeing primarily through need-based pathways, consistent with self-determination theory (Deci and Ryan, 2000; Ryan and Deci, 2017). However, the absence of consistent formal mediation testing limits inference regarding causal structure.

#### Secondary and provisional mechanisms

Additional pathways, including affective processes, behavioral regulation (e.g., need crafting), and relational processes, were observed but were less consistently specified and often modeled as outcomes or parallel predictors rather than mechanisms. Operationalization patterns further indicate that the literature relies heavily on indirect measures, with endorsement-based (35.1%) and congruence-based (20.0%) approaches dominating, and relatively fewer studies directly modeling mechanistic pathways (Appendix D).

#### Disruption–recovery asymmetry

A consistent asymmetry was observed between disruption and restoration processes.

Disruption processes (e.g., need frustration, discrepancy) were well represented and consistently associated with declines in wellbeing, whereas recovery-related processes were examined in fewer studies (n = 48) and were rarely embedded in temporally explicit models (Table 2). Cross-tabulation of mechanism type by timescale (Table 4) further indicates that mechanistic classifications are derived primarily from cross-sectional or concurrent designs, with limited integration into temporally sequenced analyses.

Overall, the evidence supports a minimal hierarchical structure, with need satisfaction as the primary pathway and other mechanisms as provisional and less consistently supported extensions.

### Risk of bias and quality summary

Quality appraisal indicates that the strongest evidence derives from intensive longitudinal (EMA/diary) designs, which allow direct estimation of within-person processes. These studies frequently employed multilevel modeling and within−/between-person decomposition, supporting robust inference regarding short-timescale associations. However, even within this category, concurrent measurement was common, limiting inference about temporal directionality. Lagged and temporally sequenced models were less frequent and less consistent.

In contrast, quantitative observational studies were typically cross-sectional or sparsely longitudinal, limiting their ability to support dynamic or causal interpretations. These studies contributed primarily to identification of associations rather than process-level mechanisms.

Theoretical and conceptual papers provided important contributions to construct clarification and mechanism specification but were not treated as empirical evidence. Across the dataset, methodological quality was predominantly moderate, reflecting trade-offs between ecological validity and limitations such as self-report measurement, incomplete temporal separation, and variability in construct operationalization. Taken together, these patterns indicate that the evidence base is strongest for short-timescale, within-person associations, but remains limited in its ability to support causal, temporal, and integrative models of alignment processes.

## Discussion

The present systematic review examined how congruence-based processes are conceptualized, operationalized, and empirically linked to short-timescale wellbeing across motivational, relational, and value-based literatures. Across 130 included studies, the findings indicate that the literature does not converge on a single construct, but instead reflects a set of related processes organized around correspondence between internal orientations and lived experience. In this respect, the review supports a multi-process structure rather than a unified construct model. Motivational processes were the most coherent and empirically developed, relational processes were beneficial but more conditional, and value-based processes were conceptually relevant but comparatively underdeveloped in temporally sensitive designs. These patterns confirm both the fragmentation and the structured overlap that motivated the review.

The results also showed that the strongest empirical evidence is concentrated in within-person, short-timescale designs, particularly diary and experience sampling studies. Across this evidence base, alignment-related processes were most consistently associated with affective and experiential indicators of wellbeing, including positive affect, vitality, engagement, and reduced strain. However, the evidence was dominated by concurrent or same-window associations, with comparatively limited support for lagged, sequential, or longer-term temporal dynamics. Thus, the review supports short-cycle covariation more strongly than extended regulatory sequencing.

A further asymmetry emerged in the mechanistic evidence. Need satisfaction and frustration were the most consistently supported pathways linking correspondence-based processes to wellbeing, especially within the motivational domain. In contrast, restoration and recovery processes were less consistently defined and less frequently modeled, even though they are central to theoretical claims about dynamic wellbeing. This asymmetry is reflected in the broader evidence base, which documents disruption more clearly than restoration and gives stronger support to misalignment-related declines than to recovery trajectories.

Taken together, these findings support the conclusion that congruence-based processes are best understood as a patterned but non-integrated process space. The literature converges on shared mechanisms and outcomes, but rarely examines motivational, relational, and value-based processes within the same empirical models. As a result, the review identifies a constrained structure of relationships rather than a validated, unified system.

### Theoretical implications

A central theoretical implication of this review is that alignment should not be treated as a single psychological construct, but as a framework for organizing functionally distinct processes. Across motivational, relational, and value-based traditions, the reviewed literature repeatedly examined forms of correspondence between internal orientations—including motives, needs, values, and identities—and lived experience (Ryan and Deci, 2000, 2017; Sheldon and Elliot, 1999; Reis et al., 2000; Kristof, 1996; Kernis and Goldman, 2006; Prentice et al., 2020). Although these traditions differ in terminology and explanatory emphasis, constructs such as autonomous motivation, self-concordance, authenticity, value congruence, moral identity, and person–environment fit all address whether behavior, goals, relationships, or contexts are experienced as consistent with psychologically significant aspects of the self (Ryan and Deci, 2000, 2017; Sheldon and Elliot, 1999; Kristof, 1996; Kernis and Goldman, 2006; Prentice et al., 2020). The findings therefore suggest that congruence is best understood not as a single construct, but as a family of related processes organized around correspondence between internal orientations and experience.

An important alternative interpretation is that the functional structure identified here primarily reflects self-determination theory, the most extensively represented tradition in the evidence base. Several observations argue against a purely SDT-derived explanation. Alignment indicators appeared across motivational, relational, fit, authenticity, and moral/value traditions; disruption and restoration processes were represented in discrepancy, fit, recovery, and crafting literatures not explicitly grounded in psychological needs theory; and constructs from multiple traditions occupied similar functional roles despite differing theoretical assumptions (Ryan and Deci, 2000, 2017; Sheldon and Elliot, 1999; Kristof, 1996; Kernis and Goldman, 2006; Prentice et al., 2020). Nevertheless, because motivational and need-based research predominated in the reviewed evidence base, the proposed framework should remain provisional until comparable evidence accumulates within relational and value-based domains. Accordingly, the framework should not be interpreted as a reformulation of self-determination theory, but as an attempt to organize recurring correspondence-based processes observed across multiple theoretical traditions.

As shown in Table 6, the framework incorporates constructs originating from self-determination, self-concordance, authenticity, person–environment fit, relational, value-based, and restoration traditions, extending beyond any single theoretical perspective.

**Table 6:** Theoretical traditions represented within the alignment framework.

Construct or theoretical tradition	Relationship to SDT	Included in alignment framework
Autonomy support	Core SDT construct	Yes
Need satisfaction	Core SDT construct	Yes
Autonomous motivation	Core SDT construct	Yes
Self-concordance	Related but distinct tradition	Yes
Authenticity	Related construct within portions of the SDT literature	Yes
Person-environment fit	Distinct theoretical tradition	Yes
Value congruence	Related construct emphasized in adjacent traditions	Yes
Moral identity	Distinct theoretical tradition	Yes
Relational responsiveness	Related but distinct tradition	Yes
Recovery/restoration processes	Limited emphasis in SDT literature	Yes

The Alignment Framework does not propose new psychological constructs. Rather, it organizes constructs originating from multiple traditions according to their functional role as contextual conditions, correspondence indicators, mechanisms, disruption processes, response processes, or restoration processes.

The review further indicates that conceptually related constructs often occupy different functional roles. Some constructs primarily index correspondence itself, such as autonomous motivation, self-concordance, authenticity, value congruence, and person–environment fit (Ryan and Deci, 2000, 2017; Sheldon and Elliot, 1999; Kristof, 1996; Kernis and Goldman, 2006; Prentice et al., 2020). Others function as contextual conditions that facilitate or constrain correspondence, including autonomy support and relational responsiveness (Deci et al., 1989; Reis et al., 2000; Legate et al., 2017). Still others function as mechanisms through which correspondence influences wellbeing, most notably psychological need satisfaction (Ryan and Deci, 2000, 2017; Reis et al., 2000). By distinguishing these roles, the alignment framework clarifies where relationships are theoretically implied and where they remain empirically untested. This distinction matters because many empirical models currently treat constructs such as autonomous motivation, psychological need satisfaction, and autonomy support as parallel predictors of wellbeing despite substantial theoretical and empirical grounds for differentiating their functions. As the review indicates, this practice risks producing structurally mis-specified models in which mechanisms are treated as independent predictors, contextual conditions are modeled as additive inputs, and functionally related processes are assumed to operate independently. The consequence is that estimated effects may reflect model specification rather than underlying psychological structure. Under the alignment framework, motivational correspondence is more appropriately treated as an indicator, need satisfaction as a mechanism, and contextual variables as antecedent or moderating conditions.

The findings also have implications for how dynamic wellbeing should be theorized. Across the reviewed literature, wellbeing was most consistently observed when behavior and context corresponded with internal orientations and declined when that correspondence was disrupted. Correspondence, in this context, refers to the extent to which behavior, goals, relationships, or contexts fit, support, or express an individual’s motives, values, needs, or identities (Ryan and Deci, 2000, 2017; Sheldon and Elliot, 1999; Reis et al., 2000; Kristof, 1996). Disruption refers to breakdowns or mismatches in these relationships, including need frustration, self-discrepancy, value incongruence, relational conflict, or environmental misfit (Higgins, 1987; Ryan and Deci, 2000, 2017; Legate et al., 2017). Although congruence-based processes are frequently described as dynamic, much of the available evidence comes from concurrent within-person associations, particularly in diary and experience-sampling studies. Such findings support co-occurrence, but they do not establish temporal ordering or causal direction, a limitation already well recognized in within-person methodology (Bolger and Laurenceau, 2013; Hamaker et al., 2015).

Accordingly, the present framework is intentionally constrained. It does not claim that the literature has already established a complete temporal sequence linking correspondence, disruption, response, and restoration. Rather, the findings suggest that disruption processes are more consistently specified and empirically supported than restoration processes.

Correspondence was most often reflected through indicators such as autonomous motivation, self-concordance, authenticity, and value congruence, whereas disruption was reflected through need frustration, discrepancy, incongruence, conflict, or unsupportive contexts (Ryan and Deci, 2000, 2017; Higgins, 1987; Legate et al., 2017; Kernis and Goldman, 2006; Prentice et al., 2020). Restoration processes, including repair, recovery, need crafting, behavioral adjustment, and re-engagement with supportive contexts, appeared less consistently and were rarely examined within temporally explicit designs. Nevertheless, the available evidence suggests that restoration was most often associated with recovery activities, opportunities for need re-satisfaction, autonomy-supportive environments, behavioral adjustment, and efforts to restore correspondence following disruption (van Hooff and Geurts, 2014; Laporte et al., 2022; Xue et al., 2022; Kujanpää and Olafsen, 2024). Across studies reporting restoration-related outcomes, recovery activities, need re-satisfaction, autonomy-supportive environments, behavioral adjustment, and re-engagement with valued activities or goals were recurrent themes. However, the reviewed studies rarely examined these factors within a common framework, making it difficult to determine which conditions reliably distinguish restoration from continued strain. Consequently, the present review can identify candidate restoration processes but cannot yet specify the contextual or individual factors that consistently predict successful restoration following disruption.

In this sense, the contribution of the framework is not the introduction of new variables, but the clarification of how existing constructs may be functionally related. The review provides a basis for integrating motivational, relational, and value-based literatures around recurring forms of correspondence while identifying important gaps in current knowledge regarding disruption, restoration, and longer-term temporal dynamics.

### Toward a dynamic alignment framework of wellbeing

The present findings support a cautious shift toward a dynamic alignment framework of wellbeing, but not a fully validated process model. Across studies, wellbeing was most consistently observed when behavior and context corresponded with internal orientations and declined when that correspondence was disrupted. Within this interpretation, alignment is not treated as a standalone explanatory variable, but as a property of relationships among variables that covary with wellbeing within persons over short timescales.

To organize these findings, the review supports a recurrent but asymmetric sequence beginning with correspondence, followed by disruption, response, and potential restoration. This sequence is not presented as an established causal chain, but as a constrained representation of the strongest recurring empirical patterns. Disruption and its immediate consequences are consistently supported, whereas restoration processes are less clearly specified and more variable. As a result, the framework adopts a disruption-dominant interpretation, in which negative deviations are more reliably observed than recovery processes. Within this structure, correspondence is most consistently indexed through autonomous motivation and need satisfaction, disruption is reflected in need frustration, discrepancy, or unsupportive contexts, and responses include behavioral and cognitive efforts such as need crafting, activity selection, or relational engagement. Response refers to behavioral, cognitive, or relational efforts that occur following disruption and are directed toward managing, compensating for, or restoring correspondence between internal orientations and lived experience. However, these latter processes are rarely embedded within temporally explicit designs, limiting inference about causal sequencing.

This interpretation is represented visually in Figure 4, which presents an event-centered conceptual model of short-term disruption and potential restoration in within-person vitality. As described in the manuscript, Figure 4 depicts fluctuations in wellbeing relative to an individual baseline following a disruption event at time *t*, followed by two possible pathways: one involving re-established correspondence and partial restoration, and the other involving continued strain under persistent misalignment. Importantly, this figure is heuristic rather than causal. It is intended to clarify the structure of the existing evidence, not to imply that a single, empirically established recovery function has already been demonstrated across studies.

**Figure 4:**
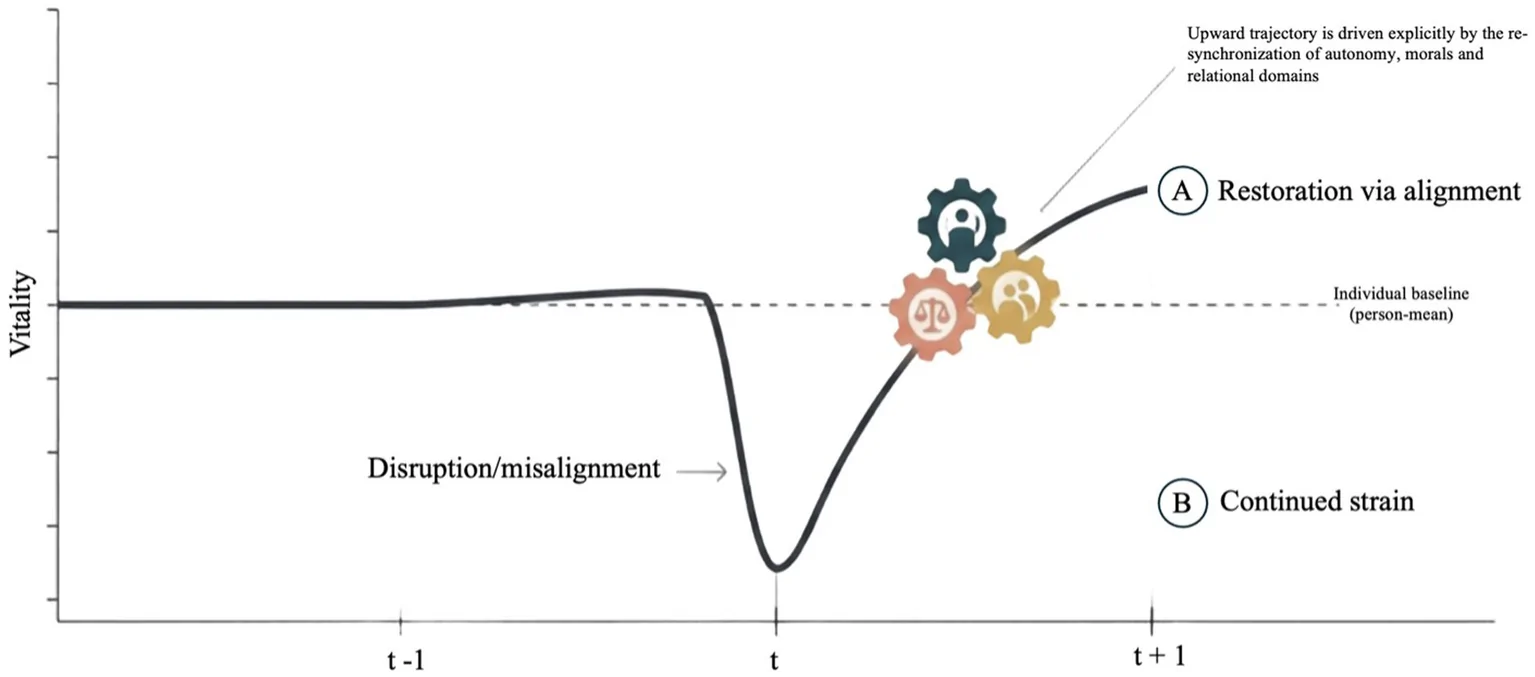
Illustrative model of short-term disruption and potential restoration in within-person vitality. The figure depicts a conceptual, illustrative trajectory of within-person vitality across short timescales. A disruption or misalignment event (*t*) is associated with a decline in vitality relative to an individual baseline (person-mean), followed by divergent potential pathways. Path A represents potential restoration associated with re-synchronization across motivational, relational, and value-based processes, whereas Path B reflects continued strain in the absence of such re-synchronization. The trajectory is intended as a heuristic representation of temporal dynamics rather than an empirically estimated function, and does not imply a single underlying causal mechanism or uniform recovery pattern across individuals or contexts.

A key implication of this framework is that the literature reveals common outcomes without demonstrated integration. Motivational processes show the strongest evidence and are most consistently linked to wellbeing through need satisfaction. Relational processes function primarily as contextual conditions shaping the enactment of internal orientations, while value-based processes remain conceptually relevant but empirically underdeveloped, particularly in within-person research. The inclusion of value-based processes in Figure 4 and in the broader framework therefore reflects their theoretical relevance but should be interpreted cautiously given the weaker temporally sensitive evidence in this domain.

### Implications for theory and research

The review identifies several priorities for future research. First, advancing this framework requires moving beyond isolated predictor models toward designs that directly evaluate the joint operation of congruence-based processes within individuals. This includes multilevel approaches examining within-person variation, tests of cross-domain interactions, and lagged or event-based analyses capable of assessing temporal sequencing. Such designs are necessary to determine whether motivational, relational, and value-based processes operate independently, conditionally, or as partially overlapping contributors to wellbeing.

Second, future work should address the imbalance between disruption and restoration evidence. The present synthesis shows that disruption is comparatively well specified and consistently associated with short-term declines in wellbeing, whereas restoration remains under-modeled and less frequently tested. This suggests a need for research that explicitly models full dynamic sequences, including disruption, response, and recovery, rather than examining these components in isolation.

Third, the field would benefit from more direct tests of construct overlap and discriminant validity. The review documents substantial conceptual redundancy among constructs such as autonomous motivation, self-concordance, need satisfaction, and broader fit-based variables, yet these are rarely compared directly within the same designs. Clarifying whether these constructs are distinct, partially overlapping, or differently positioned within a common process structure is essential for cumulative theory development.

Finally, stronger temporal inference will require designs that move beyond same-time associations. Although diary and experience sampling studies provide the strongest current evidence, many still rely on concurrent measurement, which limits conclusions about ordering and causality. Short-lag, multi-lag, and event-centered designs are therefore needed to determine whether momentary correspondence predicts later wellbeing, whether disruption triggers specific response processes, and under what conditions restoration occurs.

### Future directions

The present synthesis identifies several priorities for advancing research, particularly in relation to cross-domain integration, temporal modeling, and construct precision. A central priority concerns the role of relational processes. Constructs such as autonomy support are frequently modeled as independent predictors of wellbeing; however, the present findings suggest that these variables function more consistently as contextual conditions shaping motivational processes. Future research should therefore examine relational variables within explicitly specified pathways linking social context to motivational correspondence and need satisfaction, rather than treating them as parallel predictors.

A second priority concerns value- and moral-based processes. Although these constructs are theoretically central to correspondence-based models of wellbeing, they remain empirically underdeveloped, particularly at the within-person level. Intensive longitudinal designs are needed to assess fluctuations in value-consistent behavior, the emergence of discrepancy, and the conditions under which such discrepancies are resolved over time. Without such designs, the role of value-based processes within dynamic models of wellbeing cannot be adequately specified.

A third priority concerns the modeling of temporal dynamics. While disruption effects are consistently supported, subsequent processes, particularly behavioral and cognitive responses, are not well captured in the existing literature. Future research should explicitly model disruption–response–recovery sequences, using lagged, multi-lag, or event-based designs to determine whether, and under what conditions, restoration occurs. More broadly, the predominance of concurrent within-person findings limits temporal inference. Advancing the field will therefore require designs capable of distinguishing co-occurrence from temporally extended processes, including multilevel and time-structured approaches.

Finally, the synthesis highlights the need for direct tests of construct overlap and discriminant validity. Substantial conceptual redundancy persists among constructs such as autonomous motivation, value congruence, and relational support, yet these constructs are rarely examined within the same empirical models. Unified designs that directly compare these variables are necessary to determine whether they represent distinct processes, partially overlapping constructs, or alternative operationalizations of correspondence. Such work is essential for improving conceptual clarity and supporting cumulative theory development.

### Limitations

Several limitations constrain the interpretation of the present synthesis. First, conceptual overlap among congruence-based constructs remains unresolved. Although the alignment framework distinguishes indicators, mechanisms, and contextual conditions, these distinctionsare partly interpretive and reflect broader ambiguity in the literature. As a result, classification decisions necessarily involve theoretical judgment and may not fully resolve underlying conceptual redundancy.

Second, much of the evidence relies on proxy indicators of correspondence, particularly psychological need satisfaction and autonomous motivation. These constructs are frequently treated as direct measures of alignment despite not assessing congruence explicitly, which limits construct validity and complicates interpretation of findings.

Third, the empirical literature is heavily dominated by short-timescale, concurrent within-person designs. While these designs provide strong evidence for proximal associations, they offer limited insight into temporal sequencing and longer-term dynamics. In addition, domain representation is uneven, with strong coverage of motivational processes, moderate representation of relational processes, and comparatively limited development of value-based processes, particularly in temporally sensitive research.

Fourth, heterogeneity in construct definitions, measurement approaches, and analytic strategies precluded quantitative synthesis and necessitated an interpretive approach. Although appropriate for the aims of the review, this approach introduces subjectivity and limits replicability relative to formal meta-analytic methods. In addition, screening, extraction, and coding procedures were conducted primarily by a single reviewer. Although explicit decision rules, iterative refinement procedures, subset review, and audit trail documentation were employed to enhance consistency and transparency, classification decisions remain vulnerable to interpretive bias. Future reviews would benefit from independent multi-reviewer coding and formal assessment of coding agreement.

Finally, direct tests of cross-domain relationships remain scarce. The conditional and integrative relationships proposed in this review are therefore theoretically derived rather than empirically established, and require explicit testing in future research. In addition, reliance on aggregated coding required collapsing categories across studies, which may obscure finer-grained distinctions among constructs and processes.

## Conclusion

This review addresses fragmentation in the wellbeing literature by examining motivational, relational, and value-based constructs within a common framework centered on correspondence between internal orientations and lived experience. Across domains, wellbeing is most consistently associated with the extent to which motives, needs, and values are enacted and supported in daily contexts. Importantly, this relationship is most strongly supported at the within-person level and over short timescales, rather than as stable between-person differences.

By distinguishing indicators of correspondence, contextual conditions, and downstream mechanisms, the framework clarifies functional differences among constructs that are often treated as interchangeable. In doing so, it identifies motivational processes, particularly as specified in self-determination theory, as the most consistently supported pathway to wellbeing, while situating relational influences as contextual conditions and value-based processes as a more limited and underdeveloped extension.

The proposed dynamic alignment framework should be interpreted as a constrained synthesis rather than a fully specified model. It organizes recurring empirical patterns into a disruption-dominant structure while treating restoration processes and cross-domain relationships as provisional. Accordingly, it does not establish an integrated system, but instead specifies the conditions under which such integration can be meaningfully tested.

At the same time, the primary contribution of the framework is not the introduction of new constructs, but the imposition of constraints on how existing constructs can be validly modeled and interpreted. Without such constraints, current approaches permit theoretically inconsistent specifications in which indicators, mechanisms, and contextual conditions are treated as interchangeable predictors, leading to ambiguous or misleading conclusions regarding causal structure, temporal dynamics, and the relative importance of constructs. By enforcing role differentiation, the framework enables models that are both theoretically coherent and statistically interpretable.

The findings also help clarify why seemingly distinct constructs were included within a common congruence-based framework. Although autonomous motivation, self-concordance, authenticity, value congruence, moral identity, and person–environment fit originated from different theoretical traditions, they each describe forms of correspondence between internal orientations and lived experience (Ryan and Deci, 2000, 2017; Sheldon and Elliot, 1999; Kristof, 1996; Kernis and Goldman, 2006; Prentice et al., 2020). Similarly, autonomy support and relational responsiveness function primarily as contextual conditions that facilitate such correspondence, whereas need satisfaction functions as a mechanism through which correspondence is associated with wellbeing (Reis et al., 2000; Ryan and Deci, 2000, 2017; Legate et al., 2017). The results therefore suggest that these constructs are related not because they are identical, but because they occupy recurring positions within a broader pattern linking correspondence, disruption, and wellbeing.

## Data Availability

The original contributions presented in the study are included in the article/Supplementary material, further inquiries can be directed to the corresponding author.
