# Supplementary material for: Reclassifying congruence in wellbeing: a systematic review and alignment framework
Source: Front Psychol. 2026 Oct 1;17:1859907. doi: 10.3389/fpsyg.2026.1859907 (PMC13668872; doi:10.3389/fpsyg.2026.1859907)
Supplement: Supplementary file 1 [file Supplementary_file_1.docx]

**Appendix A: Search Strategy and Study Identification** **A1. Database Coverage**

The literature search was conducted across the following databases:

- PsycINFO
- PsycArticles
- Scopus
- Web of Science
- JSTOR
- ERIC
- Academic Search Complete
- Sociological Abstracts
- ProQuest Dissertations and Theses

Searches were supplemented by backward and forward citation tracking, as well as identification of additional records from related project searches and reference lists.

# A2. Search Structure

Search strategies were developed in accordance with the review protocol, using a layered, concept-driven approach to balance precision and coverage. Searches were structured around three core conceptual domains:

1. Alignment-related constructs

(e.g., autonomous motivation, self-concordance, value congruence, self-discrepancy, authenticity, autonomy support, responsiveness, validation)

2. Short-term well-being and recovery outcomes

(e.g., affect, mood, vitality, engagement, energy, restoration, depletion, daily or momentary well-being)

3. Within-person or short-timescale designs

(e.g., experience sampling, ecological momentary assessment [EMA], diary methods, intensive longitudinal designs, repeated measures, lagged or same-day analyses)

Primary empirical searches required the co-occurrence of all three domains to ensure inclusion of studies directly relevant to alignment processes operating within short-term timescales.

A supplementary conceptual search was conducted using alignment-related constructs combined with process-oriented terms (e.g., *dynamic*, *recovery*, *disruption*, *regulation*) to capture theoretical and mechanistic papers relevant to the review aims.

Search strategies were adapted for each database using appropriate Boolean operators, truncation, and proximity constraints to reduce retrieval of irrelevant trait-based or non-alignment literatures.

# A3. Example Search String

## Example (PsycArticles) Search completed on: March 31, 2026

(

TI(

autonom* OR "autonomous motivation" OR volition* OR self-concordan* OR "self-determination"

OR (need N3 satisfaction) OR "internal endorsement"

OR (value N3 congruen*) OR (value N3 consisten*) OR (self N3 discrepan*) OR (moral N3 identit*) OR (person N3 value N3 fit)

OR ((authentic* OR authenticity) N5 (action OR behavior OR behaviour OR values)) OR (autonomy N3 support*) OR responsiveness OR validation OR "being understood" OR "relational authenticity" OR "prosocial impact" OR "task significance"

) OR AB(

autonom* OR "autonomous motivation" OR volition* OR self-concordan* OR "self-determination"

OR (need N3 satisfaction) OR "internal endorsement"

OR (value N3 congruen*) OR (value N3 consisten*) OR (self N3 discrepan*) OR (moral N3 identit*) OR (person N3 value N3 fit)

OR ((authentic* OR authenticity) N5 (action OR behavior OR behaviour OR values)) OR (autonomy N3 support*) OR responsiveness OR validation OR "being understood" OR "relational authenticity" OR "prosocial impact" OR "task significance"

)

)

AND (

TI("well-being" OR wellbeing OR affect* OR mood OR vitality OR engagement OR energy OR "positive affect" OR "negative affect" OR restoration OR recover* OR replenish* OR depletion OR "daily well-being" OR "momentary well-being")

OR

AB("well-being" OR wellbeing OR affect* OR mood OR vitality OR engagement OR energy OR "positive affect" OR "negative affect" OR restoration OR recover* OR replenish* OR depletion OR "daily well-being" OR "momentary well-being")

) AND (

TI("experience sampling" OR "ecological momentary assessment" OR EMA OR ESM OR "daily diary" OR "daily process" OR "intensive longitudinal" OR "within-person" OR "within person" OR lagged OR momentary OR "same-day" OR "next-day" OR spillover)

OR

AB("experience sampling" OR "ecological momentary assessment" OR EMA OR ESM OR "daily diary" OR "daily process" OR "intensive longitudinal" OR "within-person" OR "within person" OR lagged OR momentary OR "same-day" OR "next-day" OR spillover)

)

# A4. Search Dates and Retrieval Counts Table A1.

*Records Identified Through Database Searching (n = 1,135)*

| **Database** | **Number of Articles Found** |
| --- | --- |
| PsycINFO | 141 |
| PsycArticle | 106 |
| ERIC | 160 |
| Sociological Abstracts & Applied Social Sciences Abstracts | 22 |
| Academic Search Complete | 35 |
| JSTOR | 177 |
| Scopus | 87 |
| Web of Science | 19 |
| ProQuest Dissertation & Theses Google Scholar (back search)  ProQuest Arts & Humanities | 15  227  146 |

# A5. Screening Flow

Following deduplication, records were screened using a two-stage PRISMA-aligned process (title/abstract screening followed by full-text review). Screening decisions were guided by explicit inclusion criteria requiring:

- a relevant alignment-related construct
- a short-term or momentary well-being/recovery outcome
- within-person or short-timescale relevance The final sample comprised 130 studies**.**

# A6. Study Classification and Appraisal Framework

Included studies were classified into four design categories using predefined decision rules:

| **Category** | **Decision Rule** | **Appraisal Emphasis** |
| --- | --- | --- |
| Quantitative observational | One- or few-wave surveys, sparse panel designs, multilevel cross-sectional studies, or non-dense repeated-session designs; experimental studies were included here when no better classification fit | Sampling appropriateness; measurement validity; confounding control; statistical rigor |

| **Category** | **Decision Rule** | **Appraisal Emphasis** |
| --- | --- | --- |
| Intensive longitudinal / diary / EMA | Dense within-person repeated assessments across days or weeks, including EMA/ESM and diary designs; mixed studies were classified here when intensive longitudinal data were primary | Observational criteria plus sampling density; compliance/retention; within–between separation; predictor–outcome timing; lag structure; timescale justification |
| Qualitative | No eligible qualitative primary studies identified in the dataset | CASP criteria would have been applied if present |
| Theoretical / conceptual | Theory papers, narrative reviews, editorials, and meta-analyses without primary data | Conceptual clarity; definitional precision; temporal mechanisms; cross-domain integration; relevance |

# A7. Distribution of Study Designs

The final dataset was dominated by intensive longitudinal research:

- Intensive longitudinal / diary / EMA: 100 studies
- Quantitative observational: 18 studies
- Theoretical / conceptual: 12 studies
- Qualitative**:** 0 studies

This distribution reflects the review’s focus on within-person, short-timescale processes, while also incorporating supporting observational and conceptual literatures.

# A8. Overview of Methodological Characteristics

Across the included studies:

- 106 studies explicitly modeled within-person processes using centering or decomposition techniques
- 98 studies included temporally structured analyses, including same-day repeated assessments, temporally separated observations, or lagged models; 55 of these included explicitly lagged analyses.
- 10 studies reported compliance or retention metrics
- A subset of observational studies relied on cross-sectional or single-wave designs

Overall methodological quality was predominantly moderate, with fewer high-quality studies and a small number of low-quality designs.

# A9. Summary of Evidence Base

The evidence base is characterized by:

- Strong representation of intensive longitudinal designs, providing direct insight into within-person alignment processes
- More limited observational evidence, primarily useful for mapping associations and contextual factors
- A smaller but important body of theoretical work**,** contributing to conceptual clarification and mechanism development

This structure supports a synthesis strategy that prioritizes within-person temporal evidence while integrating observational and conceptual contributions in a complementary manner.

# Appendix B: Quality Appraisal Framework

**B1. Rationale**

Because the review integrates heterogeneous study types, including intensive longitudinal (diary/EMA), quantitative observational, and theoretical/conceptual work, a single risk-of-bias tool was not appropriate. Instead, appraisal was conducted using a design-specific framework aligned to the classification rules used in the dataset.

Studies were first categorized based on design features rather than labels alone, using explicit decision rules (e.g., dense within-person sampling vs. sparse-wave designs). Appraisal criteria were then tailored to each category to ensure that evaluation captured the methodological features most relevant to the review’s focus on short-term alignment processes and well-being.

This approach follows best practice for mixed conceptual–empirical syntheses and ensures that within-person process evidence, between-person associations, and theoretical contributions are evaluated on appropriate terms.

# B2. Study Classification and Decision Rules

Studies were classified into four categories using predefined decision rules:

- Quantitative observational

One/few-wave survey or panel designs, sparse repeated follow-up, multilevel cross-sectional analyses, or non-dense repeated-session designs. Experimental studies were mapped here when no better classification fit the available schema.

- Intensive longitudinal (diary/EMA)

Dense within-person repeated assessments across days or weeks, including experience sampling or momentary designs. Mixed-design papers were classified here when the diary/EMA component provided the primary evidence relevant to the review.

- Qualitative

No eligible qualitative primary studies were identified in the dataset.

- Theoretical / conceptual

Theory papers, editorials, narrative reviews, and meta-analyses/secondary syntheses not based on a single primary dataset.

This classification structure directly informed the appraisal criteria applied to each study type.

# B3. Observational Study Criteria

Quantitative observational studies were evaluated using JBI/NIH-style criteria, with emphasis on:

- Sampling appropriateness (representativeness, recruitment, size)
- Measurement validity (validated instruments, reliability, construct alignment)
- Control of confounding (covariates, alternative explanations)
- Statistical analysis rigor (model appropriateness, transparency, robustness)

Across the dataset, this body of evidence was heavily weighted toward cross-sectional or sparse-wave designs, limiting its ability to support causal or dynamic process claims.

# B4. Intensive Longitudinal (Diary/EMA) Criteria

Intensive longitudinal studies were evaluated using the observational criteria above, plus design-specific dimensions critical for within-person inference:

- Sampling density (frequency and duration of repeated assessments)
- Compliance and retention reporting
- Within- vs. between-person separation (e.g., centering, multilevel modeling)
- Temporal alignment of predictors and outcomes
- Lag structure and temporal sequencing
- Justification of timescale relative to theoretical processes

Across the dataset, this category formed the dominant evidence base (n = 100 studies). Nearly all studies implemented within-/between-person decomposition (n = 100), and most included lagged or temporally separated models (n = 98). However, same-day or concurrent measurement remained common, limiting directional inference in many cases.

# B5. Theoretical / Conceptual Study Evaluation

Conceptual and theoretical papers were evaluated using a structured theoretical relevance framework, focusing on:

- Conceptual clarity
- Definitional precision
- Explicit specification of temporal mechanisms
- Cross-domain integration (motivational, relational, value-based processes)
- Relevance to the review question

These papers (n = 12) were not assessed for risk of bias but instead for their ability to clarify constructs, specify mechanisms, and integrate domains relevant to alignment processes.

# B6. Quality Rating Scheme

All empirical studies were assigned an overall quality rating:

| **Rating** | **Description** |
| --- | --- |
| High | Strong design, valid measurement, appropriate modeling, and clear temporal alignment |
| Moderate | Adequate design with limitations (e.g., self-report reliance, concurrent timing, limited lag structure) |
| Low | Substantial limitations affecting interpretability |

Across the dataset:

- High quality: 34 studies
- Moderate quality: 92 studies
- Low quality: 4 studies

Overall, methodological quality was predominantly moderate, reflecting recurring trade-offs between ecological validity (e.g., diary designs) and limitations such as self-report measurement and incomplete temporal separation.

# B7. Cross-Study Appraisal Insights

## Balance of Evidence

The evidence base is strongly dominated by intensive longitudinal designs (n = 100), with fewer quantitative observational studies (n = 18) and a smaller conceptual segment (n = 12).

## Strength of Within-Person Evidence

The strongest empirical support comes from diary/EMA studies, which directly estimate within-person covariation in motivation, need satisfaction, and well-being. Many of these studies employ multilevel modeling and decomposition techniques, supporting robust inference about short-term alignment processes.

However, concurrent timing remains common, meaning that many findings support temporal alignment and co-occurrence more strongly than directional causality.

# Limitations of Between-Person Evidence

Quantitative observational studies are valuable for identifying associations and broader population patterns, but are typically cross-sectional or sparsely longitudinal, limiting their ability to support claims about dynamic processes such as disruption or recovery.

# Patterns in Methodological Quality

Higher-quality studies tend to include:

- Dense repeated sampling
- Explicit within-/between-person separation
- Temporal ordering or lagged models

Lower-confidence studies are typically limited by:

- Same-time measurement
- Short sampling windows
- Single-item or self-report measures
- Convenience samples

# Role of Conceptual Work

Theoretical papers contribute most when they:

- Clearly define alignment-related constructs
- Specify temporal mechanisms
- Integrate across domains

Their contribution is weaker when constructs remain implicit or temporality is underspecified.

# B8. Implications for Evidence Weighting

Findings were interpreted using a hierarchical weighting approach:

- Highest weight:

Intensive longitudinal studies with dense sampling, within-/between-person separation, and temporal sequencing

- Moderate weight:

Diary studies with concurrent measurement or limited lag structure

- Lower weight:

Cross-sectional or sparse-wave observational studies

- Conceptual contribution:

Used to inform theory and mechanism, but not treated as empirical evidence

Accordingly:

- Cross-sectional findings were not used to support causal claims
- Temporally aligned diary evidence was prioritized for conclusions about short-term processes
- Theory-driven claims were explicitly framed as conceptual rather than empirical

# B9. Methods Justification

The use of design-specific appraisal criteria combined with a theoretical relevance framework reflects the structure of the dataset and the aims of the review.

- Observational studies were appraised using JBI/NIH-style criteria
- Intensive longitudinal studies were appraised using extended criteria capturing within-person dynamics and temporal structure
- Conceptual papers were evaluated based on their explanatory and integrative contribution

This integrated approach ensures that conclusions are methodologically grounded, appropriately weighted, and aligned with the strengths and limitations of the underlying evidence base

# Appendix C: Coding Framework and Variable Definitions

**C1. Coding Dimensions**

All included studies were systematically coded using a structured framework designed to capture both methodological characteristics and theoretical function within the review model. Coding was conducted at the level of the primary analysis relevant to the review question.

Each study was coded on the following dimensions:

- Study design

Categorized using the same decision rules as Appendix B (intensive longitudinal/diary/EMA, quantitative observational, theoretical/conceptual), based on the structure and density of data collection rather than author labels.

- Domain

Constructs were coded into one or more conceptual domains reflecting the focus of the review:

- *Motivational* (e.g., autonomous vs. controlled motivation, goal regulation)
- *Relational* (e.g., autonomy support, interpersonal processes)
- *Value-based* (e.g., value congruence, self-concordance, identity alignment)
- Timescale of analysis

Coding captured the temporal structure of the evidence:

- *Short-term (concurrent or same-window)*
- *Lagged / temporally separated* (e.g., prior-day, cross-lagged, sequential within-day)
- *Cross-sectional / sparse-wave*
- Construct type

Each variable extracted from a study was coded according to its conceptual role in the model (see C2).

- Mechanism classification

Where applicable, constructs were further coded according to the specific process pathway they represented (e.g., need-based, affective, regulatory), enabling cross-study synthesis of mechanisms.

This multi-dimensional coding structure enabled integration of heterogeneous designs while preserving distinctions between within-person processes, between-person associations, and theoretical constructs.

# C2. Functional Classification Framework

To support synthesis across diverse literatures, all constructs were classified according to their functional role within the alignment process model. This framework distinguishes between what is being aligned, how alignment operates, and the conditions under which it occurs.

| **Category** | **Definition** | **Examples** |
| --- | --- | --- |
| Indicators | Direct measures of correspondence, alignment, or congruence between internal states, goals, or contexts | Autonomous motivation; value congruence; self-concordance |
| Mechanisms | Processes through which alignment influences outcomes, typically representing proximal psychological or affective pathways | Need satisfaction/frustration; affect; subjective vitality; regulatory processes |
| Contextual conditions | Factors that shape, enable, or constrain the expression of alignment processes | Autonomy support; social context; environmental affordances |

This functional classification allowed constructs from different theoretical traditions to be mapped onto a common explanatory structure, facilitating comparison across studies and domains.

# C3. Decision Rules

To ensure consistency and transparency in coding, the following decision rules were applied:

- Primary functional role assignment

Each construct was classified according to its *dominant theoretical function* within the study, even when it could plausibly serve multiple roles.

- Prioritization of within-person evidence

When studies reported both within-person and between-person effects, coding prioritized the within-person component, as this was most directly relevant to the review’s focus on short-term processes.

- Handling of mixed or multi-study papers

For studies with multiple designs or components, coding was based on the component most relevant to the review question, consistent with the classification approach used in Appendix B.

- Temporal classification rules

Timescale coding reflected the actual analytic structure (e.g., lagged models vs. same-time associations), rather than the nominal study design.

- Iterative refinement of ambiguous cases

Ambiguous constructs or borderline classifications were resolved through iterative rule refinement, ensuring internal consistency across the dataset.

- Audit trail documentation

All coding decisions, including reclassifications and edge cases, were documented in an audit trail, providing transparency and reproducibility.

# C4. Integration with Evidence Synthesis

The coding framework was designed to directly support cross-study synthesis by:

- Linking study design and timescale to evidentiary strength
- Mapping constructs onto a shared functional model
- Enabling comparison of mechanisms across domains
- Distinguishing process-level evidence (within-person) from associational evidence (between-person)

This structure allowed findings to be synthesized in a way that reflects both conceptual coherence and methodological rigor, ensuring that conclusions are grounded in the type and strength of evidence available.

# Appendix D: Supplementary Quantitative Analyses D1. Temporal Distribution

To characterize the temporal structure of the evidence base, all coded observations were classified according to timescale. Results indicate that the literature is dominated by cross-sectional and short-term designs, with relatively limited use of lagged or temporally extended analyses.

| **Timescale** | **Count** | **Percentage** |
| --- | --- | --- |
| Short-term (within-day or same-window) | 208 | 40.6% |
| Lagged / temporally separated | 10 | 2.0% |
| Cross-sectional / sparse-wave | 294 | 57.4% |

These findings indicate that although a substantial portion of studies capture within-person or short-timescale processes, the majority of evidence remains cross-sectional or non-temporally structured, and only a small proportion explicitly models lagged or sequential relationships.

**D2. Domain × Timescale Association**

To examine whether temporal structure varies across conceptual domains, a chi-square test of independence was conducted. Results indicated a significant association between domain and timescale:

χ²(9) = 64.04, *p* < .001, Cramér’s V = .57

This effect size suggests a substantial association, indicating that different domains (motivational, relational, value-based) are not evenly represented across timescales. In particular, motivational constructs are more frequently examined using short-term or within-person designs, whereas relational and value-based constructs are more often studied using cross-sectional or less temporally resolved approaches.

# D3. Mechanism Distribution

To assess how alignment-related processes are operationalized across studies, constructs were coded according to mechanism type. The distribution of mechanism classifications is shown below:

| **Mechanism Type** | **Percentage** |
| --- | --- |
| Endorsement-based processes | 35.1% |

| **Mechanism Type** | **Percentage** |
| --- | --- |
| Congruence-based measures | 20.0% |
| Fit / discrepancy-based processes | 14.0% |
| Direct mechanism modeling | 10.2% |
| Support-based processes | 7.7% |
| Inferred proxy measures | 8.1% |
| Combined support/fit measures | 4.9% |

These results indicate that the literature is dominated by indirect operationalizations, particularly endorsement and congruence-based measures, with comparatively fewer studies directly modeling mechanistic pathways linking alignment processes to well-being outcomes.

# D4. Structural Model Summary

To explore structural relationships among coded components of the framework, a linear model was estimated examining associations between construct layers.

The model was statistically significant:

F(2, 91) = 14.32, *p* < .001, R² = .24

This model indicates that variation in outcome-layer representation is associated with variation in upstream construct categories, although these relationships should be interpreted as descriptive associations within the coded dataset, rather than as causal effects.

# D5. Interpretation Note

These quantitative analyses are based on study-level coding rather than pooled effect sizes and are therefore intended to support descriptive synthesis and pattern identification, not meta-analytic inference.

Accordingly:

- Statistical results should be interpreted as reflecting distributional patterns in the literature, not effect magnitudes
- Associations identified here do not imply causal relationships between constructs
- Findings are used to inform weighting of evidence and guide conceptual integration, consistent with the review’s interpretive synthesis approach

# Appendix E

**Primary Full-Text Exclusion Categories**

In response to reviewer feedback and consistent with PRISMA 2020 recommendations, the primary reasons for exclusion at the full-text review stage are summarized below. The categories below represent the principal reasons used for exclusion during full-text eligibility assessment.

# Primary Exclusion Categories

1. **No alignment-related, congruence-based, or correspondence-based construct examined**

Studies did not investigate constructs relevant to correspondence between internal orientations (e.g., motives, needs, values, identities) and lived experience.

# 2. No short-timescale or within-person outcome

Studies focused exclusively on between-person associations, cross-sectional outcomes, or long-term outcomes without examination of daily, momentary, diary, or experience-sampling processes.

# 3. Trait-level or global well-being only

Studies assessed stable or global indicators of well-being but did not include temporally sensitive outcomes relevant to the review objectives.

# 4. Insufficient relevance to the review questions

Studies addressed related topics but did not contribute directly to understanding alignment-related processes, correspondence, disruption, response, restoration, or short-timescale well-being.

# 5. Methodological or reporting limitations

Studies lacked sufficient methodological detail, conceptual clarity, or outcome reporting to permit evaluation within the present synthesis.
